# Treatment of Aortic and Iliac Artery Occlusion by Catheter Thrombolysis Combined with Catheter Thrombectomy and Aortic Bifurcation Endovascular Stent Reconstruction

**DOI:** 10.1155/2021/6084226

**Published:** 2021-11-17

**Authors:** Xinyu Zhao, Delang Liu, Chaowen Yu, Yong Sun, Shiyuan Chen

**Affiliations:** Department of Vascular Surgery, First Affiliated Hospital of Bengbu Medical College, Bengbu, Anhui Province, China

## Abstract

Aortoiliac occlusive disease (AIOD) is an occlusive disease of the infrarenal aorta and iliac arteries usually caused by stenosis or occlusion at the end of the abdominal aorta-common iliac artery. Herein, we reported a case of Trans-Atlantic Inter-Society Consensus- (TASC-) D AIOD with pale, cool, and intangible dorsalis pedis artery treated with catheter thrombolysis combined with catheter thrombectomy and aortic bifurcation endovascular stent reconstruction, which proved to be safe, effective, and minimally invasive approach. In the present paper, we discussed the physical and imaging manifestations, as well as treatments.

## 1. Introduction

Aortoiliac occlusive disease (AIOD) refers to the ischemic disease of lower extremities or pelvic tissue and organs caused by occlusion at the end of the abdominal aorta-bifurcation of the main iliac artery. The clinical manifestations include the disappearance of femoral artery pulsation, intermittent claudication, and impotence, which in some severe cases can cause distal limb gangrene. The most common cause of AIOD is atherosclerosis.

In the present case, due to atherosclerosis, bilateral common iliac, internal iliac, external iliac artery, and right anterior tibial artery proximal lumen occlusion were not treated in time, resulting in aortic and bilateral iliac artery thrombosis. The patient was then treated with catheter thrombolysis combined with catheter thrombectomy and aortic bifurcation endovascular stent reconstruction, which proved to be safe and effective approach.

## 2. Case Presentation

**Figure 1:**
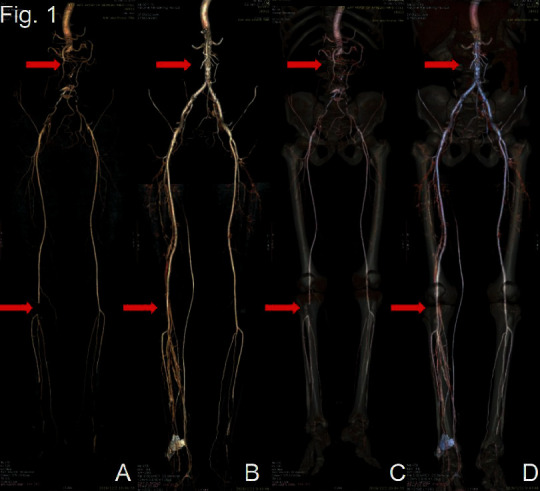
CTA before operation ((a) and (c)) after the operation ((b) and (d)). The red arrow points to the location of the lesion before and after the operation.

A 54-year-old male patient was admitted to our center on December 2, 2019. The patient presented with chills, numbness, and intermittent claudication in both lower limbs. Fontaine stage was IIa. The specialist physical examination revealed that the patient's pulsation of the left dorsalis pedis artery was weak, and the right dorsalis pedis artery was not palpable. Computed tomographic angiography (CTA) showed signs of filling defect in the proximal lumen of the common iliac, internal and external iliac arteries, and the right anterior tibial artery. They were found to have “continuity interruption” at the end of the abdominal aorta, and irregular stenosis of the local lumen of the bilateral femoral artery, deep femoral artery, popliteal artery, left anterior and posterior tibial artery, and peroneal artery was observed (Figures 1(a) and 1(c)).

During the first hospitalization, the patient refused surgical treatment and was given anticoagulation and vasodilation therapy for one week. After discharge, the patient was treated with antiplatelet aggregation and anticoagulation by oral administration of cilostazol and salgrel axetil. During the medication period, the patient developed ischemic rest pain (Fontaine stage III).

**Figure 2:**
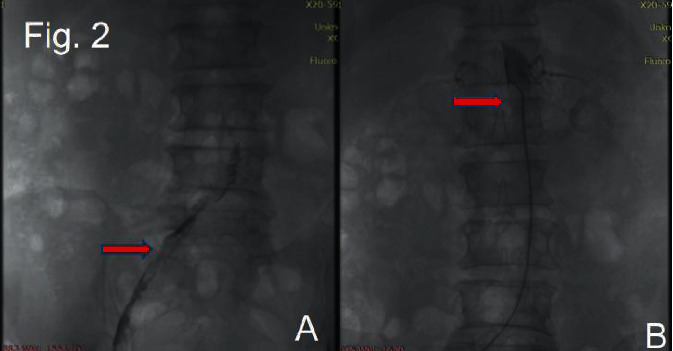
The first DSA image. Occlusion of the right iliac artery and multiple thromboses in the wall (a). Occlusion of the abdominal aorta at the level of the right renal artery (b).

On January 8, 2020, the patient was readmitted to the hospital. Specialist physical examination revealed pale skin on both feet, the skin was also warm and cool, and the dorsalis pedis arteries were not palpable. Digital subtraction angiography (DSA) revealed that the right iliac artery was occluded, multiple thrombi were attached to the wall, and the abdominal aorta was occluded from the right renal artery level (Figure 2).

**Figure 3:**
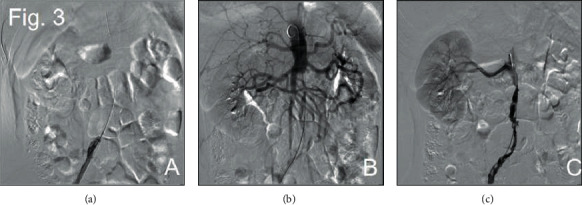
The second DSA image. Thrombolysis catheter blockage, partial development of right external iliac artery (a). Thrombolysis in the proximal renal abdominal aorta (b). Replacement of thrombolytic catheter angiography (c).

**Figure 4:**
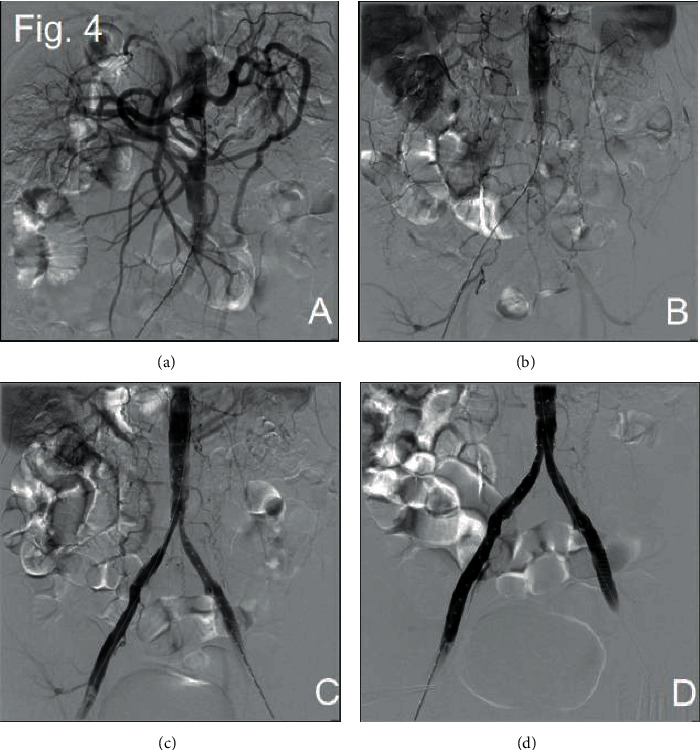
The third DSA image. Angiography before and after thrombectomy ((a) and (b)). Angiography before and after balloon dilatation ((c) and (d)).

**Figure 5:**
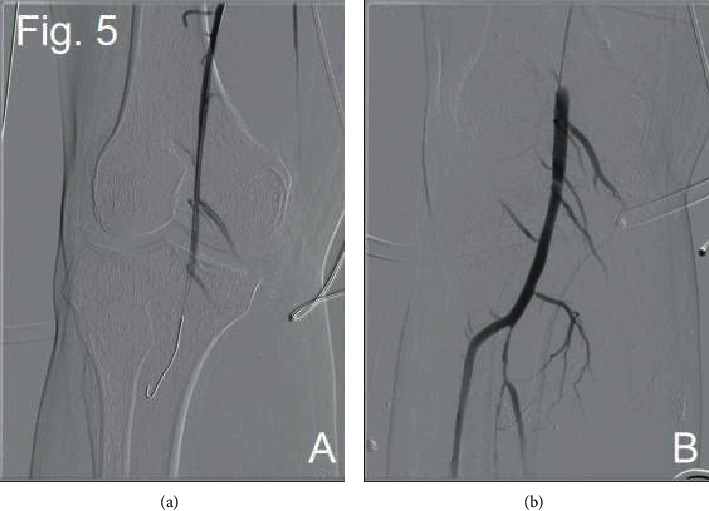
Angiography of right popliteal artery before and after thrombectomy ((a) and (b)).

Through three operations that were performed at our center, we successfully opened the abdominal aorta and bilateral iliac arteries to improve the symptoms of lower limb ischemia. During the first procedure, we punctured the right femoral artery, successfully passed through the occlusive segment of the right iliac artery, and placed the end tip of the 5F 20cm UNIFUSE thrombolytic catheter into the abdominal aorta at the level of the right renal artery. Thrombolysis with 200000u urokinase was continuously given after operation. Two days later, we performed a second DSA operation. Thrombolytic catheter angiography showed that the right external iliac artery was partially developed, but the distal abdominal aorta was not seen. The possibility of blockage was considered at the tip end of the thrombolytic catheter, and the thrombolysis angiography showed that the thrombus in the proximal renal abdominal aorta was dissolved more than before. Replacement of 5F 20 cm UNIFUSE thrombolysis catheter continued thrombolysis for 24 hours (Figure 3). During the third procedure, we used thrombectomy combined with covered endovascular reconstruction of the aortic bifurcation (CERAB). The angiography of the right femoral artery showed a large filling defect below the opening of the renal artery of the abdominal aorta, the development of the right iliac artery was unobstructed, and the left iliac artery was not seen (Figure 4(a)). The 14 mm balloon was placed above the renal artery, and the balloon was filled and pulled to the end of the abdominal aorta to move the filling defect downward (Figure 4(b)). The 4F Fogarty balloon embolization tube was implanted into the end of the abdominal aorta, the arterial embolus was removed about 10 cm, and the thrombus was distally removed. The embolus of the left iliac artery was removed by the same method. The pulsation of bilateral femoral arteries was palpable after thrombectomy. The lower segment of the abdominal aorta was implanted with Medtronic ENDURANT EN LW 1616C95EE self-expanding covered stent, and the upper end was located below the opening of the right renal artery. Angiography showed that the blood flow in the stent of the abdominal aorta was unobstructed, and there was no contrast medium extravasation and filling defect. Two self-expanding covered stents (Bard Fluency 8 mm∗80 mm) were implanted in both common iliac arteries, where the proximal end of the two stents was located in the abdominal aortic stent, overlapping some 15 mm. Angiography showed that the blood flow in the abdominal aorta and bilateral iliac arteries was unobstructed, and the orifice of the bilateral iliac arteries was narrowed. The stenotic segments of bilateral iliac arteries were dilated with 8 mm balloon, and the angiographic results showed improved blood flow than before (Figures 4(c) and 4(d)). Finally, we placed 4F Fogarty thrombectomy balloon into the right popliteal artery and removed the artery 5 cm embolus. The angiography showed that the blood flow of the right popliteal artery was smooth (Figure 5).

After the operation, we examined the patient and found that the skin temperature in both feet recovered, and the bilateral dorsalis pedis arteries were palpable. Reexamination of CTA revealed that the abdominal aorta, bilateral common iliac, internal and external iliac arteries, bilateral femoral arteries, deep femoral arteries, and popliteal arteries were functioning well (Figures 1(b) and 1(d)).

After operation, the patient was orally given aspirin, 100 mg qd, Rivassaban 2.5 mg bid [1]. Also, the blood coagulation function of the patient was monitored; his prothrombin and international standard ratio were in the ideal range.

## 3. Discussion

Over recent years, the incidence of AIOD has been increasing year by year [2, 3]. AIOD risk factors include advanced age, smoking, obesity, diabetes, hypertension, hyperlipidemia, chronic renal insufficiency, and homocysteinemia [4]. If AIOD develops into chronic limb-threatening ischemia (CLTI), the risk of myocardial infarction, stroke, and death increases three-fold [5]. The patient reported in the present study had TASC-D type of main iliac artery occlusive disease according to TASC-II classification, and surgical treatment was recommended as the first treatment choice [4]. The total incidence of complications after AIOD surgical treatment has been reported to be about 17% to 32% [6].

AIOD treatment has gradually evolved from traditional surgical bypass surgeries to intracavitary surgery with less trauma, fewer complications, and rapid recovery [7]. We chose catheterization thrombolysis combined with catheter thrombectomy, CERAB. In this case, the signs at the first visit were mild, and the symptoms significantly aggravated after one month, suggesting that insufficient anticoagulation may lead to thrombosis of the main iliac artery. In the first angiography, there was a long segment occlusion in the lumen of the right iliac artery and fresh thrombus in the wall. Blind balloon dilatation and stenting may lead to thrombus shedding, failure of opening, and similar, eventually resulting in adverse consequences. We used vertebral artery catheter combined with hard slippery guide wire to successfully pass through the occlusive segment of iliac artery and insert thrombolytic catheter. After two thrombolysis, the aortic occlusive segment adjacent to the renal artery disappeared, which in turn reduced the level of stent placement and the possibility of stent affecting the renal artery. The diameter of the abdominal aorta of the patient was large, and there was no matching thrombectomy catheter. We used a 14 mm balloon instead of a thrombectomy catheter and successfully removed the aortic-iliac artery thrombus. Considering that the patient had long aortic-bilateral iliac artery stenosis, we used CERAB [8], a technique with a high medium- and long-term patency rate in the reconstruction of complex AIOD [9, 10]. After bilateral femoral artery angiography, the path map was made to determine the opening of the bilateral internal iliac artery, after which the bilateral common iliac artery stent was implanted into the lower segment of the abdominal aorta. The use of covered stents can effectively reduce arterial perforation, rupture, acute or chronic bleeding, and avoid intimal hyperplasia and in-stent restenosis. On the other hand, it can avoid stenosis and thrombosis caused by mural thrombus shedding of occlusive disease [11, 12]. The use of covered stents can also increase the risk of covering collateral vessels or internal iliac arteries [11]. Finally, we used balloons to dilate the abdominal aorta-double iliac artery stents so as to protect the contralateral iliac artery and further avoid embolism caused by dissection, plaque displacement, and shedding.

## 4. Conclusion

Herein, we described a case of a patient with TASC-D aortic and iliac artery occlusion who experienced significant improvement in lower limb ischemia after three operations. We believe that catheter thrombolysis combined with catheter thrombectomy and covered endovascular reconstruction of the aortic bifurcation is a safe, effective, and minimally invasive method for the treatment of long-segment aortic iliac artery occlusion.

## References

[1] Bonaca M. P., Bauersachs R. M., Anand S. S. (2020). Rivaroxaban in peripheral artery disease after revascularization. *The New England Journal of Medicine*.

[2] Fowkes F. G., Rudan D., Rudan I. (2013). Comparison of global estimates of prevalence and risk factors for peripheral artery disease in 2000 and 2010: a systematic review and analysis. *Lancet*.

[3] Song P., Rudan D., Wang M., Chang X., Rudan I. (2019). National and subnational estimation of the prevalence of peripheral artery disease (PAD) in China: a systematic review and meta-analysis. *Journal of Global Health*.

[4] Norgren L., Hiatt W. R., Dormandy J. A., Nehler M. R., Harris K. A., Fowkes F. G. R. (2007). Inter-society consensus for the management of peripheral arterial disease (TASC II). *Journal of Vascular Surgery*.

[5] Rooke T. W., Hirsch A. T., Misra S. (2011). 2011 ACCF/AHA focused update of the guideline for the management of patients with peripheral artery disease (updating the 2005 guideline). *Vascular Medicine*.

[6] Menard M. T., Belkin M. (2013). *Aortoliac disease: direct reconstruction//Cronenwett JL, Johnston KW. Rutherford’s vascular surgery*.

[7] Lun Y., Zhang J., Wu X. (2015). Comparison of midterm outcomes between surgical treatment and endovascular reconstruction for chronic infrarenal aortoiliac occlusion. *Journal of Vascular and Interventional Radiology*.

[8] Grimme F. A. B., Goverde P. C. J. M., Verbruggen P. J. E. M., Zeebregts C. J., Reijnen M. M. P. J. (2015). First results of the covered endovascular reconstruction of the aortic bifurcation (CERAB) technique for aortoiliac occlusive disease. *Journal of Vascular Surgery*.

[9] Mwipatayi B. P., Sharma S., Daneshmand A. (2016). Durability of the balloon-expandable covered versus bare-metal stents in the covered versus balloon expandable stent trial (COBEST) for the treatment of aortoiliac occlusive disease. *Journal of Vascular Surgery*.

[10] Shen C., Zhang Y., Qu C., Fang J., Liu X., Teng L. (2020). Outcomes of Total Aortoiliac Revascularization for TASC-II C&D Lesion with Kissing Self-Expanding Covered Stents. *Annals of Vascular Surgery*.

[11] Mwipatayi B. P., Thomas S., Wong J. (2011). A comparison of covered vs bare expandable stents for the treatment of aortoiliac occlusive disease. *Journal of Vascular Surgery*.

[12] Sabri S. S., Choudhri A., Orgera G. (2010). Outcomes of covered kissing stent placement compared with bare metal stent placement in the treatment of atherosclerotic occlusive disease at the aortic bifurcation. *Journal of Vascular and Interventional Radiology*.

